# Symmetry based assembly of a 2 dimensional protein lattice

**DOI:** 10.1371/journal.pone.0174485

**Published:** 2017-04-18

**Authors:** Sandra Poulos, Sayeh Agah, Nikardi Jallah, Salem Faham

**Affiliations:** Department of Molecular Physiology and Biological Physics, University of Virginia School of Medicine, Charlottesville, Virginia, United States; Centro Nacional de Biotecnologia, SPAIN

## Abstract

The design of proteins that self-assemble into higher order architectures is of great interest due to their potential application in nanotechnology. Specifically, the self-assembly of proteins into ordered lattices is of special interest to the field of structural biology. Here we designed a 2 dimensional (2D) protein lattice using a fusion of a tandem repeat of three TelSAM domains (TTT) to the Ferric uptake regulator (FUR) domain. We determined the structure of the designed (TTT-FUR) fusion protein to 2.3 Å by X-ray crystallographic methods. In agreement with the design, a 2D lattice composed of TelSAM fibers interdigitated by the FUR domain was observed. As expected, the fusion of a tandem repeat of three TelSAM domains formed 2_1_ screw axis, and the self-assembly of the ordered oligomer was under pH control. We demonstrated that the fusion of TTT to a domain having a 2-fold symmetry, such as the FUR domain, can produce an ordered 2D lattice. The TTT-FUR system combines features from the rotational symmetry matching approach with the oligomer driven crystallization method. This TTT-FUR fusion was amenable to X-ray crystallographic methods, and is a promising crystallization chaperone.

## Introduction

Proteins that self-assemble into higher order structures have garnered much interest due to their potential applications in nanotechnology such as sensors [1,2], vaccine development [3,4], diagnostics [5,6], and drug delivery [7,8]. Additionally, self-assembled proteins can be used as scaffolds for templating inorganic materials [9,10,11,12,13,14], which can have a variety of applications in nanoelectronic [15,16,17], plasmonic [18,19,20], magnetic [21], biomedical [22,23], and catalytic fields [23,24]. Efforts have been directed toward the design of proteins that self-assemble, and various architectures have been pursued such as fibers [25], nanocages [26,27], and lattices [28]. Engineering proteins into ordered lattices is of special interest to structural biologists in the fields of electron microscopy (EM) and X-ray crystallography.

Two approaches have been applied successfully for the design of two dimensional protein arrays. These are: (1) the matching rotational symmetry approach [29], and (2) computational optimization of protein-protein interfaces [28]. In both cases, the success of the design was observed only by EM techniques. Protein structure determination by X-ray crystallographic methods relies on obtaining highly ordered lattices, which can be a critical bottle neck. Protein fusions have been used to assist in crystallization by utilizing protein domains that have been described as crystallization chaperones [30,31,32,33]. Ordered two dimensional (2D) protein arrays can be potentially utilized as platforms for the production of ordered three dimensional crystals. However, this requires the arrays to be amenable to crystallographic techniques, not just EM methods. This is challenging since proteins that self-assemble into large oligomeric complexes tend to precipitate rapidly, and crystal growth typically requires higher solubility for the specimen of interest. In order to overcome this challenge, we are utilizing pH as a trigger to control the oligomerization process.

Here, we report a novel approach for the design of two dimensional protein lattices that we confirmed by X-ray crystallography to high resolution. This approach combines features from the “polymer driven crystallization” method [34], with the “rotational symmetry matching” method [29], which was used successfully to generate a 2D lattice by fusion of aminolevulinic acid dehydrogenase which has D4 symmetry to a streptavidin/streptag system which has D2 symmetry. This 2D lattice was observed by EM [29]. The “polymer driven crystallization” method is based on the human TelSAM (Tel: Translocation Ets Leukemia, SAM: Sterile Alpha Motif) domain. Tel is a member of the ETS family of transcriptional regulators which is frequently involved in human leukemias as the result of specific chromosomal translocations [35]. The Sterile Alpha Motif (SAM) is a protein interaction motif found in a wide variety of eukaryotic proteins that are involved in many biological processes such as protein-protein, protein-RNA, and protein-lipid interactions [36]. It has been shown that in some cases the SAM domain self oligomerizes, and in other cases it can bind to a variety of non-SAM domain proteins [37].

The SAM domain of Tel (TelSAM) forms an oligomer with a 6_5_ helical symmetry [38]. However, mutating residue Valine-80 to glutamate (V80E) renders the oligomeric assembly pH dependent. Therefore, pH can be used as a trigger to control the oligomerization process. Residue 80 resides at the center of what otherwise would be a largely hydrophobic oligomeric interface [38,39]. It has been shown that the V80E mutation does not cause significant structural changes at the interface compared to wtTelSAM structure [39]. It was proposed that the hydrophilic nature of the glutamate is what renders TelSAM soluble at high pH by interfering with the overall hydrophobic nature of the oligomeric interface [38]. Upon protonation of the glutamate at lower pH, it is no longer sufficiently hydrophilic to prevent the association of the TelSAM molecules. This mutated form (V80E) offers attractive features that can be exploited for use as a crystallization chaperone, and it has previously been demonstrated that TelSAM is indeed a promising crystallization chaperone [34]. These previous experiments demonstrated that: (1) TelSAM protein fusions did not hinder protein expression or folding, and a number of crystals were obtained, (2) The pH trigger worked as expected, and importantly (3) the tandem repeat of two TelSAM domains adopted the intended structure (PDB code 2QAR). Specifically, the fusion of two TelSAM domains maintained the helical polymeric nature with fewer units for each repeat turn. Thus, instead of 60° degrees rotation per unit, 120° degrees rotation per unit was obtained, and the 6_5_ screw symmetry was transformed into 3_2_ screw symmetry.

**Fig 1:**
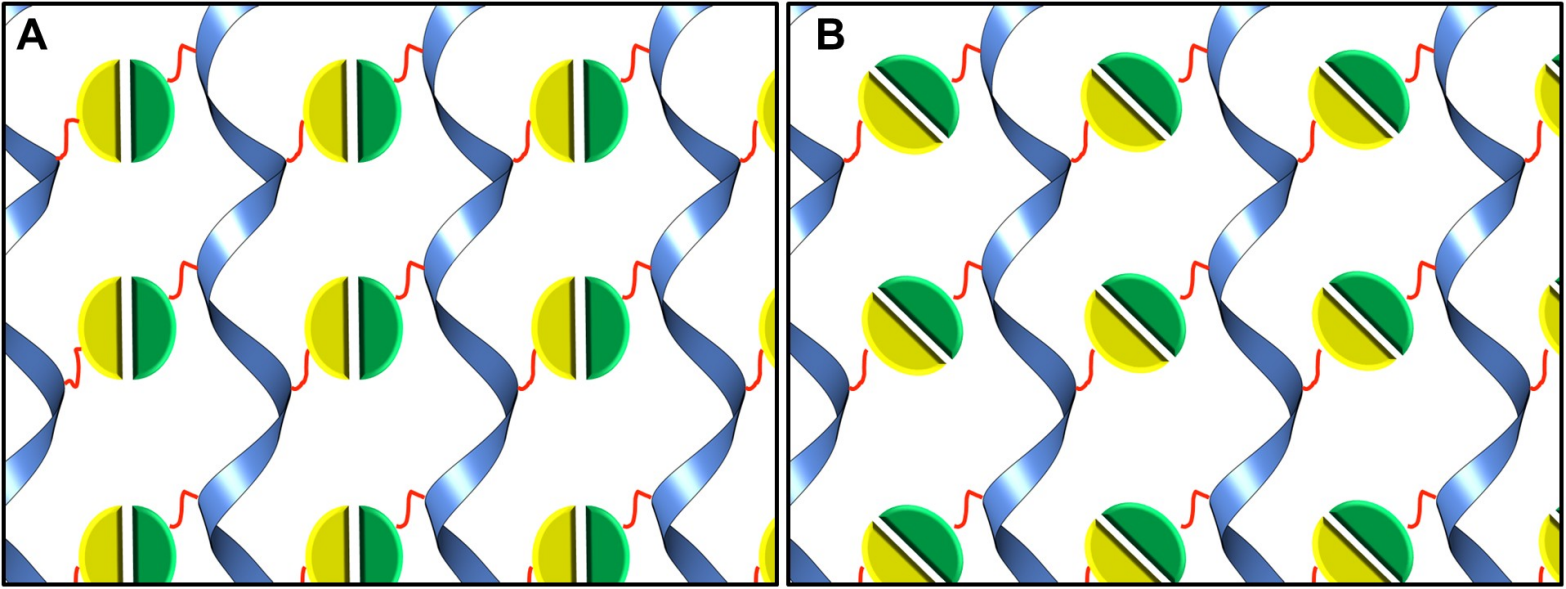
Design of the 2D lattice. The 2-fold symmetry axis for the n-FUR domain is shown to be able to adopt multiple conformations. In (A) the 2-fold axis is shown parallel to the TelSAM helical axis, and (B) shows how the n-FUR domain can adopt a different orientation. The TelSAM oligomer is shown as a blue ribbon. The n-FUR domains are shown in green and yellow, and the flexible linker is shown in red.

We build on these prior experiences in order to develop an alternate and an improved system with more favorable features. Here we utilized a tandem repeat of three TelSAM domains instead of two. With three TelSAM domains (TTT) a 180° degrees rotation per unit would be expected, which should form oligomers with each single filament having a 2_1_ screw axis symmetry. We attempt to use the 2_1_ symmetry as a backbone to form a 2D lattice by fusing a protein domain with a tendency to form a 2-fold symmetry to the 2_1_ screw axis, as shown in Fig 1A and 1B.

Fusion of TTT to a dimeric protein can lead to rapid self-assembly and precipitation of the sample (unpublished results). In order to avoid aggregation by rapid self-assembly of the resulting construct, we selected the N-terminal domain of the ferric uptake regulator (FUR) protein from *E*. *coli*. The N-terminal domain of FUR (n-FUR; residues 1 to 83) was shown to be monomeric in solution, and yet formed dimers in the crystal structure [40]. These n-FUR dimers were mediated by cadmium ions (PDB code: 2FU4). Therefore, the fusion of TTT with n-FUR would be expected to be soluble at high pH and in the absence of cadmium. A flexible linker was used to connect the n-FUR domain to TTT, in order to allow the n-FUR domain to sample many orientations and select a favorable position without restricting the orientation of the 2-fold axis (Fig 1B). Our goal for the 2-D lattice design was that the interactions between the TelSAM oligomers be entirely mediated by the n-FUR domains as shown in Fig 1A and 1B.

## Materials and methods

### Lattice design

**Fig 2:**
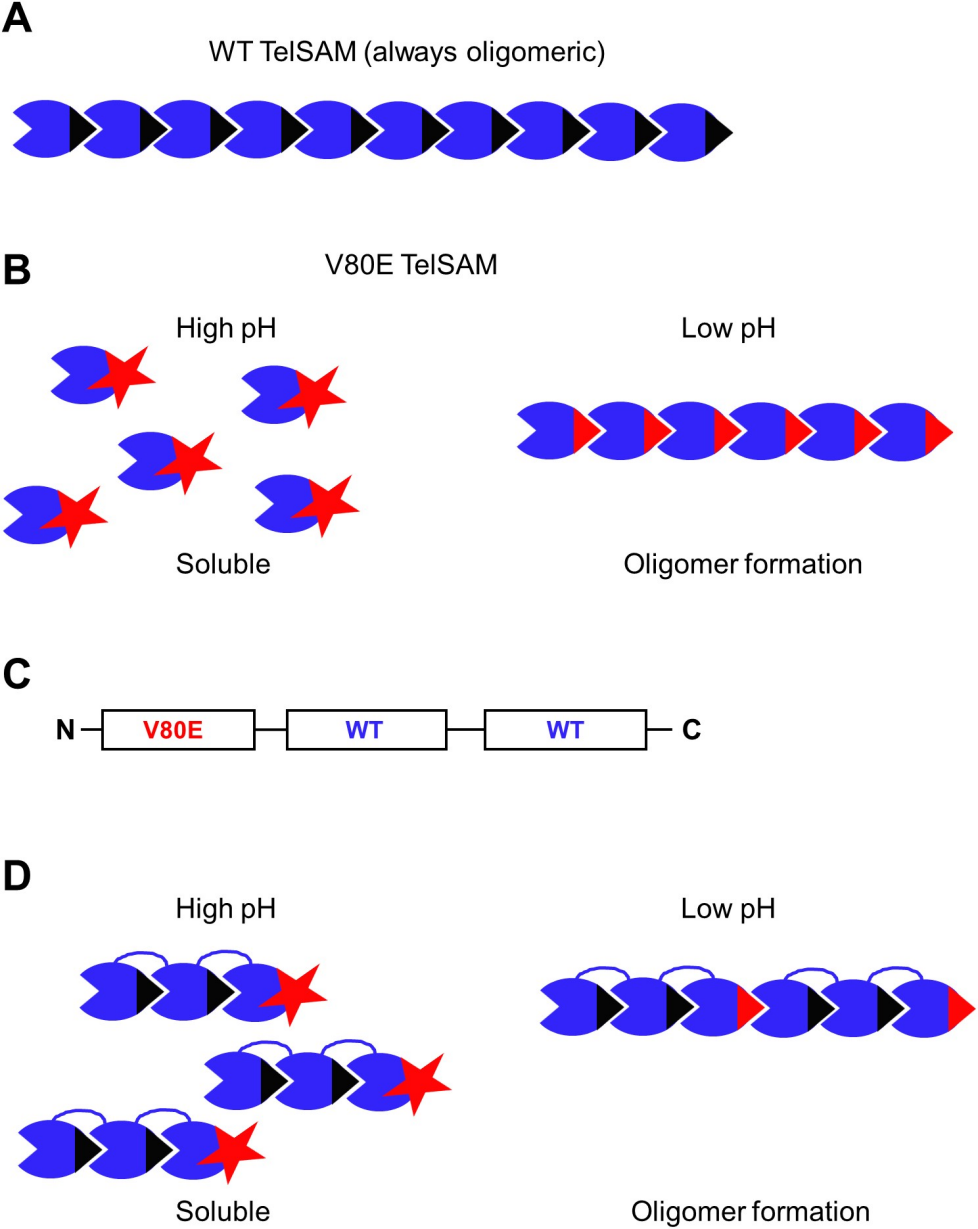
The order of the TelSAM domains and the effect of pH on solubility. The behavior of monomeric WT TelSAM (A), and V80E TelSAM (B) as a function of pH. (C) The designed order of the tandem repeat of three TelSAM domains. This order results in having the V80E on the external surface of the protein and the two internal interfaces with WT residues. (D) The predicted behavior of the fusion of the designed TTT as a function of pH, as a result of having the V80E on the external surface. Valine at position 80 is shown as a black triangle. A glutamate at position 80 is highlighted in red. At high pH the glutamate is shown as a star that promotes monomeric behavior. At low pH the glutamate is shown as a red triangle that can promote oligomerization.

Three TelSAM domains were fused to a single n-FUR domain. The order of the TelSAM domains in this fusion is V80E, WT, WT, followed by the n-FUR domain. Thus the protein domains have the following order: V80E-TelSAM → wtTelSAM → wtTelSAM → n-FUR (V80E-WT-WT-FUR) as shown in Fig 2. With this order of domains, the internal TelSAM interfaces would be the same as the wtTelSAM interface, however the external oligomeric interface presents the V80E mutation (Fig 2). This design was selected in order to ensure improved solubility of our construct, yet maintain the rigidity of the internal interfaces. Thus, the association of V80E-WT-WT-FUR with other molecules is pH dependent, whereas the internal interfaces of TelSAM are always formed (Fig 2).

### Cloning

TELSAM fusion clones were prepared by standard PCR and double digest and ligation procedures. First, the n-FUR domain was added to a tandem repeat of two SAM domains. Then, a third SAM domain was subcloned in between the second SAM domain and the n-FUR domain, which included a flexible GGGSGGGS linker to connect the TelSAM domains to the n-FUR domain.

### Protein expression, purification and crystallization

Protein expression was carried out in *E*. *coli* Top10 cells in LB containing 100 μg/mL ampicillin at 37°C. The cells were induced for 3 hours with 0.02% (w/v) L-arabinose when OD_600_ reached 0.7. The cell pellets were resuspended in buffer A containing (50 mM Tris pH 8.7, 250 mM NaCl, 5% (v/v) glycerol, and 1 mM PMSF). The cells were then disrupted by two passes through an M110-P microfluidizer (Microfluidics) at 20k psi, and centrifuged in a Beckman JA-20 rotor for 30 min at 14K rpm. The supernatant was combined with 2 mL of Ni-NTA affinity resin and stirred at 4°C for 1 hour. The resin was applied to a gravity flow column and washed with buffer A containing increasing concentrations of imidazole. The washes consisted of 30 mL of buffer A containing 10 mM imidazole, followed by 30 mL containing 30 mM imidazole, and finally 10 mL with 50 mM imidazole. The protein was eluted in 10 mL of buffer-A containing 250 mM imidazole. The eluted protein was transferred to a 10K MWCO SpectraPor Dialysis Membrane and buffer exchanged to 50 mM Tris pH 8.7 and 250 mM NaCl. The protein was concentrated in a 10K MWCO Amicon Ultracel centrifugal filter unit. The protein concentration prior to setting up crystallization conditions was 8 mg/mL. The protein was crystallized at 17°C using the hanging drop vapor diffusion method by mixing 1 μL of the protein with an equal volume of the well solution which contained 100 mM Tris pH 8.0 and 3.5 M NaCl.

### Protein precipitation curves

We tested the solubility of the protein as a function of pH and evaluated the effect of cadmium. The solubility of the protein was tested using several buffers with pHs ranging from 4.5 to 9.0, and the effect of adding 5mM cadmium chloride was examined. The solubility of 0.4 mM protein was monitored by measuring the absorbance at 400 nm on a SpectraMax M5 spectrophotometer (Molecular Devices).

### Structure determination

Crystals were flash frozen into liquid nitrogen and the data was collected at the Advance Photon Source (APS) at the ID beamline 22. The data was processed to 2.3 Å using the program HKL2000 [41]. Phases were obtained by molecular replacement with the program Phaser [42] using TelSAM and the n-FUR domain as models (pdb codes 1JI7 and 2FU4, respectively). All four domains were found by Phaser [42] (S1 File and S2 File). Model building and structural comparisons were performed using COOT [43], and refinement was carried out in Refmac [44]. The calculations of the interface surface areas were carried out using PISA [45].

#### Protein data bank accession code

The structure and data have been deposited in the PDB with the accession code 5L0P.

## Results and discussion

### Protein expression and solubility

**Fig 3:**
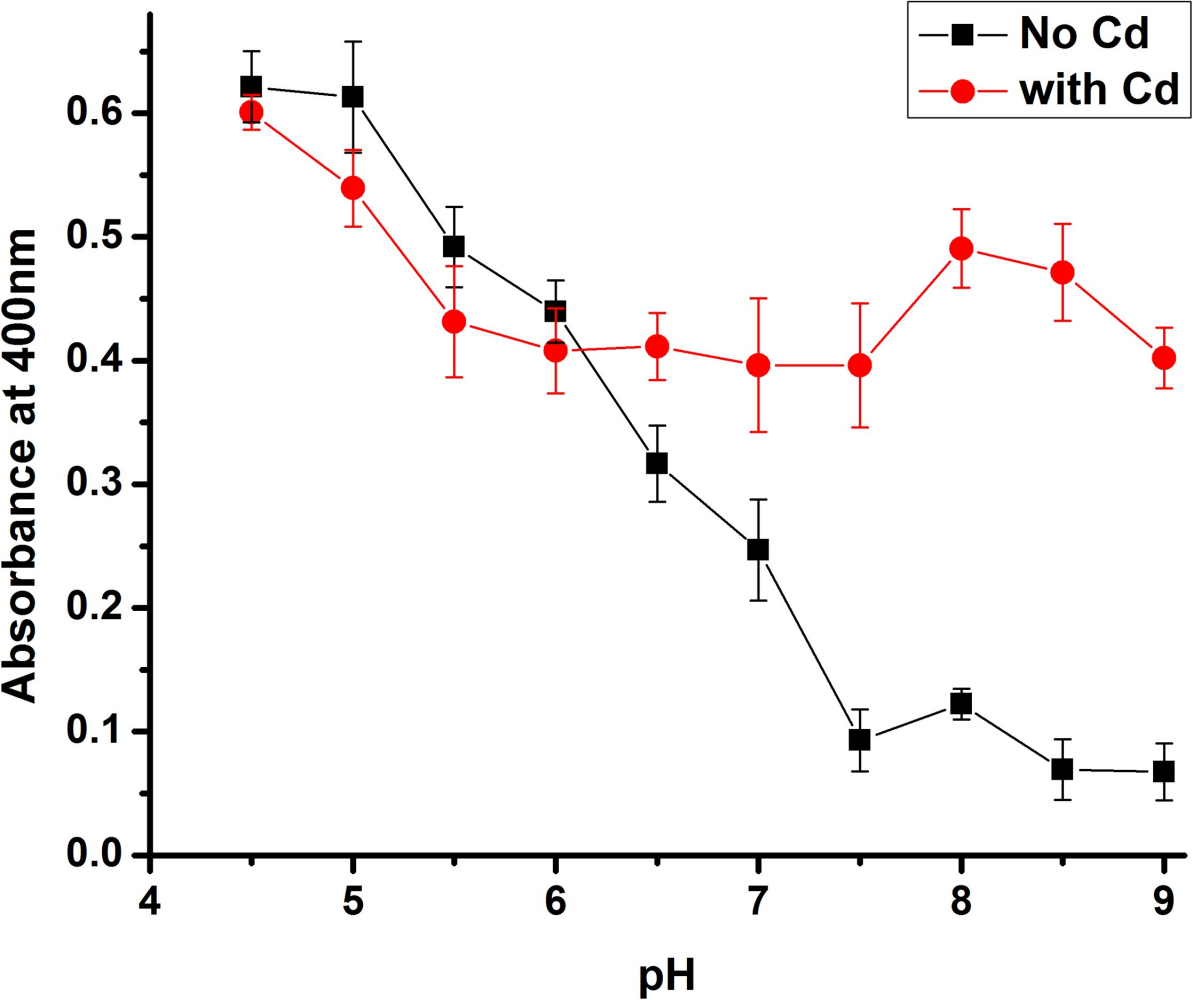
Precipitation curve. The effect of pH and cadmium on solubility. In the absence of cadmium (black squares), protein solubility is pH dependent. In the presence of cadmium (red circles), the protein is always oligomeric.

The V80E-WT-WT-FUR (TTT-FUR) construct expressed well, and was soluble at high pH (to at least 15 mg/ml). The precipitation curve demonstrated that the solubility of the (TTT-FUR) fusion protein is dependent on pH as expected. A higher absorbance is observed at lower pH, indicating the formation of large complexes. This is consistent with the V80E-TelSAM domain forming an oligomer at lower pH (Fig 2). Therefore, this result supports that the V80E mutation being present on the external surface of the protein as designed. Additionally, we observed that cadmium leads to rapid precipitation of the protein regardless of the pH (Fig 3).

### Crystallization and structure description

**Fig 4:**
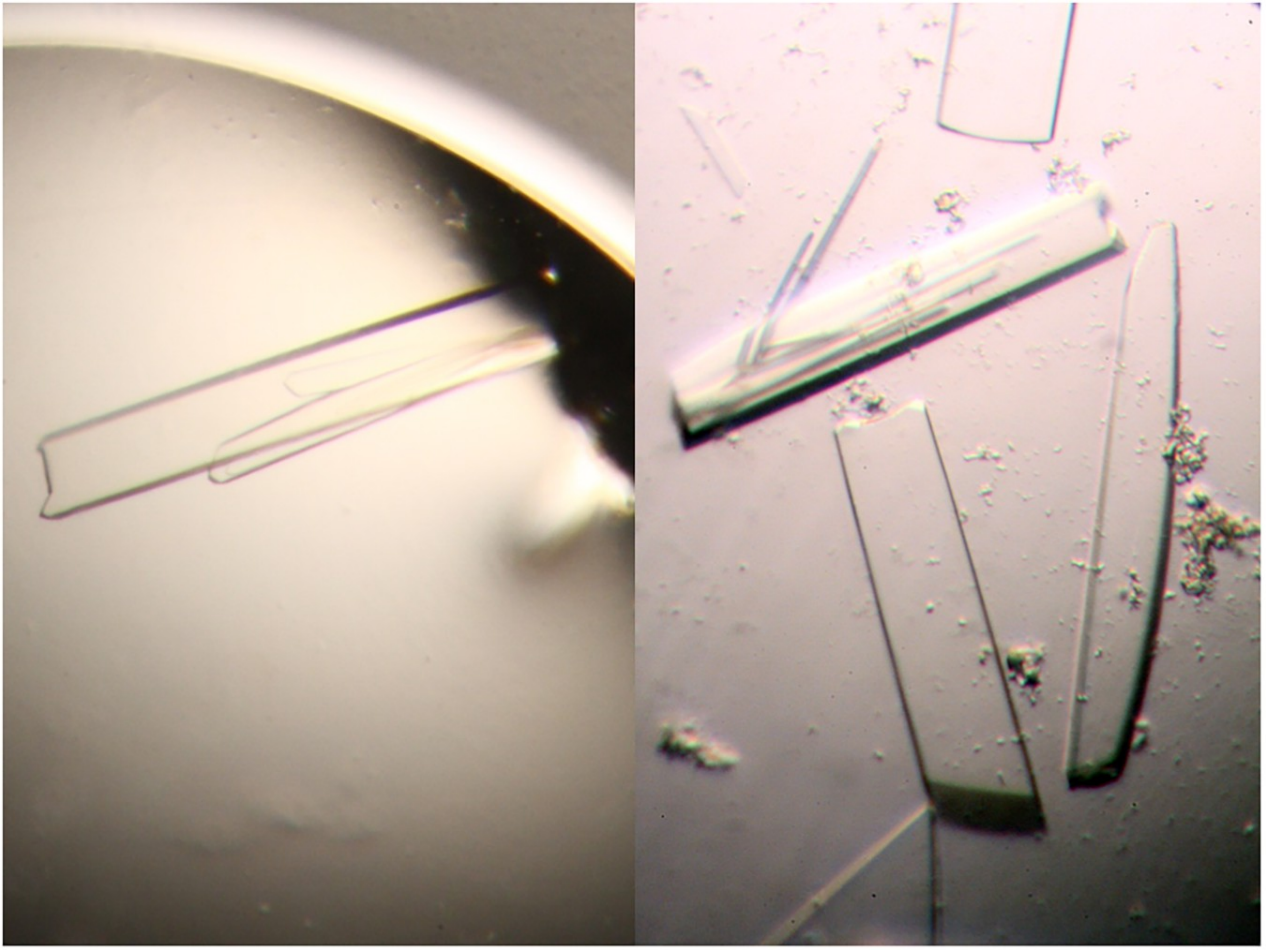
Pictures of the TTT-FUR crystals.

Crystallization screens were carried out both in the presence and absence of cadmium. Crystallization trials in the presence of cadmium precipitated rapidly, in agreement with the precipitation curves (Fig 3). A more varied behavior (soluble vs precipitated) was observed in the drops without cadmium, and crystals were identified in the trays in the absence of cadmium. Plate crystals grew to a size of 0.7x0.15x0.03 mm (Fig 4). We determined the structure of the TTT-FUR fusion protein to 2.3 Å by X-ray crystallography (Table 1). One molecule was found in the asymmetric unit, and all four protein domains (3 TelSAM domains, and 1 n-FUR domain) were identified by molecular replacement using Phaser.

**Table 1:** Data collection and refinement statistics*.

Space group	C2
Resolution range (Å)	50.0–2.30 (2.35–2.30)
Cell a, b, c (Å)	131.31, 54.83, 88.48
Alpha, beta, gamma (°)	90.0, 128.5, 90.0
Completeness (%)	93.9 (68.7)
Redundancy	4.8 (3.5)
Rmerge (%)	9.2 (28.3)
I/Sigma	15.9 (3.7)
CC ½	-92.2
Number of unique reflections measured	20782
Number of reflections used in refinement	19724
Rwork/Rfree (%)	19.82/23.56 (24.0/31.9)
Bonds RMS (Å)	0.019
Angles RMS (°)	1.935
Protein atoms	2555
Solvent atoms	120
Mean B factor (Å2)	19.28
Protein atoms	19.09
Solvent atoms	23.33
Solvent content (%)/Matthews Coeff	61.07/3.16

* Number in parenthesis corresponds to the highest resolution shell (2.35–2.30) Å.

The loops that link these domains were disordered and not visible in the electron density. Having flexible loops was a part of the design, thus this result is not a surprise. Similarly, in previous TelSAM tandem fusions, the loops were also disordered [34].

**Fig 5:**
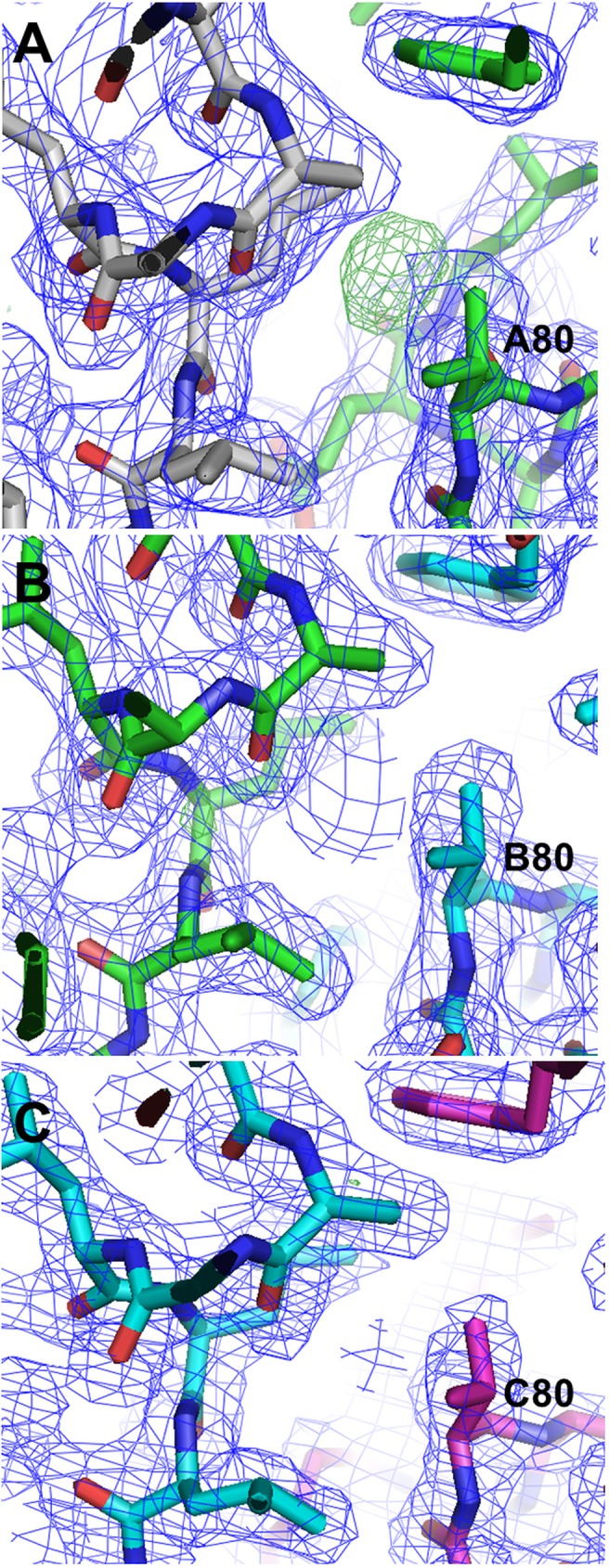
Difference electron density map (Fo-Fc) contoured at 3.0 sigma at residue 80 for all three TelSAM domains. This was calculated in the early stages of model building, with valine built at position 80 for all three TelSAM domains. The excess density indicates which of the three domains has the glutamate mutation at position 80. The first TelSAM is colored in green, the second is colored in cyan, and the third is colored in magenta. The symmetry related molecule is colored in gray.

In order to determine which of the TelSAM domains contains the mutated Glu residue at position 80, all three domains were initially modeled as valines. Difference map densities helped determine clearly which domain had additional density as would be expected for a glutamate at this position. These initial electron difference density maps are shown in Fig 5, which clearly identifies the TelSAM molecule with the V80E mutation as indicated by the amount of excess positive electron density. The identification of this mutation allowed us to place this molecule first in the sequence in accordance with the design, with the V80E TelSAM first in sequence, followed by two WT TelSAM molecules, and ending with the n-FUR domain. As expected, the structures of the internal TelSAM-TelSAM interfaces are similar to the external one formed by V80E (Fig 5). The temperature factors showed that all four domains were well ordered. The average temperature factors were 17.6, 17.8, 14.9, and 26.5 Å^2^ for the three TelSAM domains and the n-FUR domain, respectively.

### Structural comparisons

**Fig 6:**
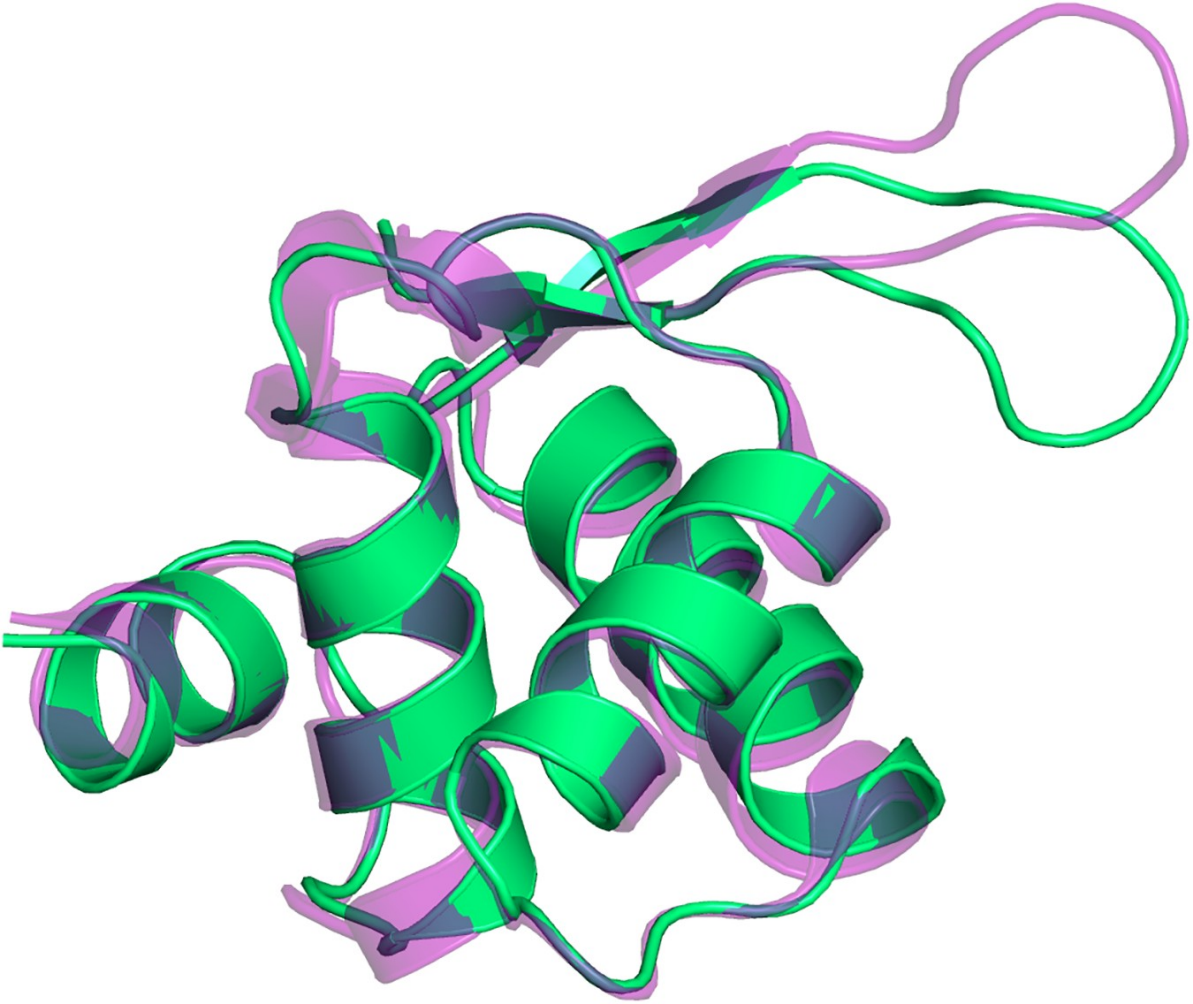
Structural comparison of the n-FUR domain. Superposition of the observed n-Fur domain (green) onto the previously determined structure (semi-transparent purple. PDB code: 2FU4).

The structures of the three TelSAM domains are similar to each other with Cα rmsd values of 0.57 Å, 0.66 Å, and 0.44 Å between the A and B chains, A and C chains, and B and C chains, respectively. The structures of the TelSAM domains are also similar to the previously determined structures. Superposition of the TelSAM domains onto the A chain of the original TelSAM structure (pdb code 1JI7) generates the following Cα RMSD values: 0.73 Å for chain A, 0.49 Å for chain B, and 0.33 Å for chain C. The RMSD values for n-FUR domain compared to the A and B chains of the previously determined structure of n-FUR (pdb code 2FU4) are 0.88 Å, and 0.95 Å, respectively. The largest change for the n-FUR domain is observed at the loop consisting of residues 72–78 as shown in Fig 6.

### Higher order structure

The organization of the 3TelSAM portion of the fusion achieved the expected structure, an oligomer with 180° degree rotation per unit that forms a 2_1_ screw axis, which can also be described as one dimensional crystals or fibers. The observed structure for the TelSAM oligomer in this structure is similar to the original TelSAM oligomeric structure (PDB code 1JI7). A repeat distance of 54.8 Å is observed in this structure vs. 52.7 Å in the original TelSAM structure. The average interface surface area per monomer between the TelSAM domains is 622.8 Å^2^ in this structure vs. 624.8 Å^2^ in the original structure. In our structure, there is one molecule in the asymmetric unit, which contains three TelSAM domains. Similarly, three TelSAM molecules were present in the asymmetric unit in the original TelSAM structure (pdb code 1JI7). If the three TelSAM domains from the original structure are treated as a single molecule and compared to our structure, an overall Cα RMSD of 1.59 Å is obtained. These results indicate structural similarities between these two structures, regarding the TelSAM monomers as well as the TelSAM oligomers.

**Fig 7:**
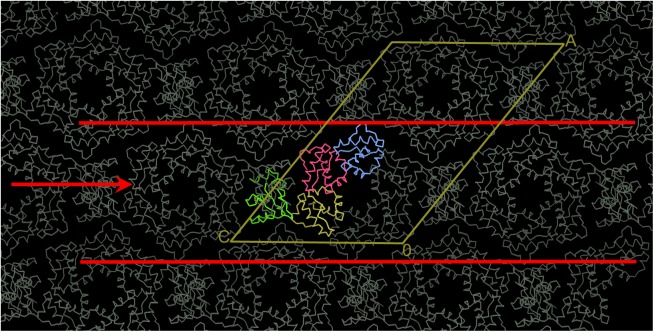
The observed 3 dimensional crystal packing, with a top-down view of the TelSAM oligomers. The TelSAM domains are shown in yellow, red, and blue, and the FUR domain is shown in green. The symmetry related molecules are shown in gray. The unit cell is shown in yellow. The crystal packing can be described as stacking of 2D crystals. The arrow between the red lines points out the 2D lattice. This view is looking down the Y axis. The unit cell dimensions are (131.3, 54.8, 88.5) Å, and the angles are (90.0°, 128.5°, 90.0°)

**Fig 8:**
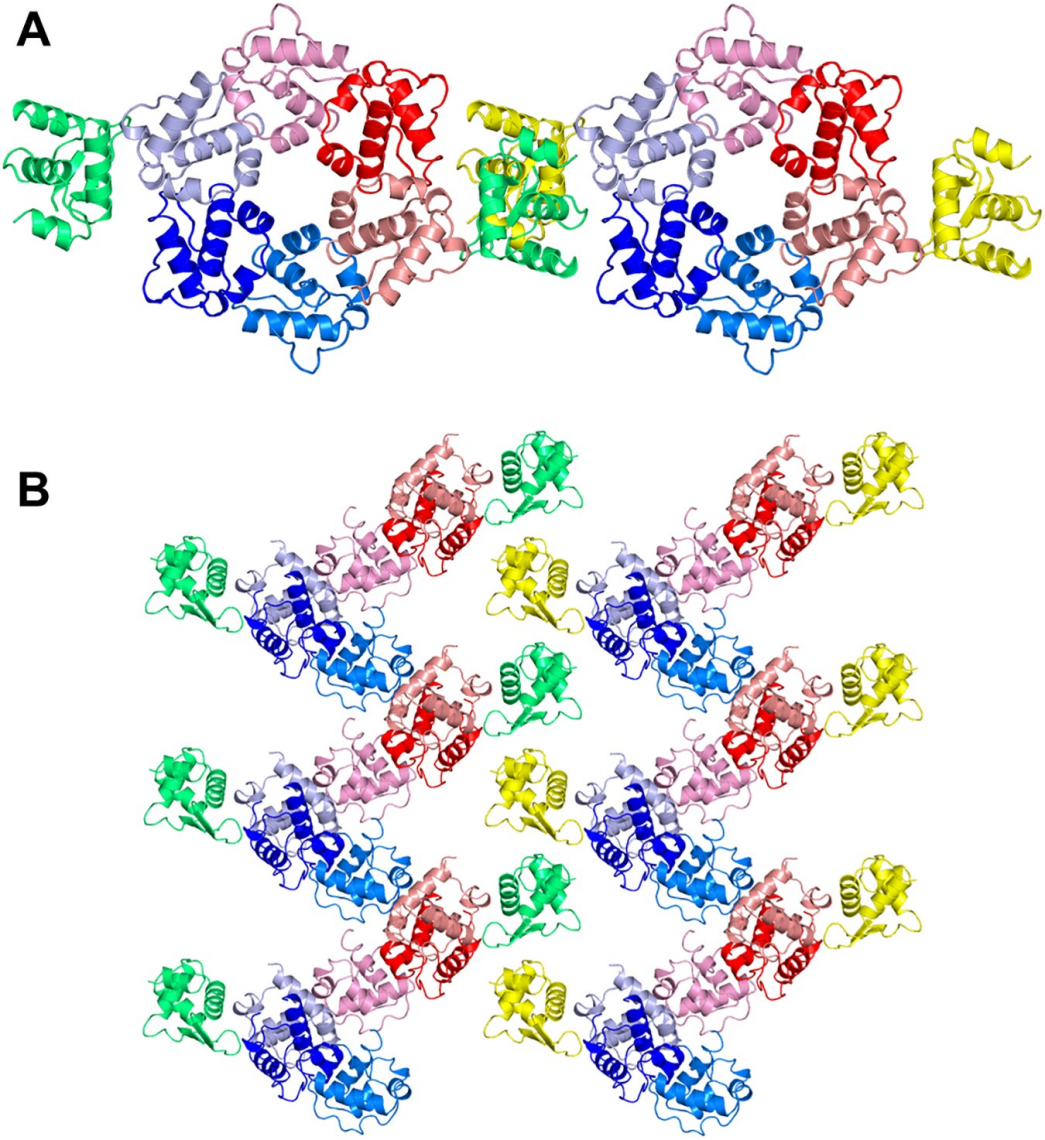
The observed 2D crystal packing. One set of TTT domains are shown in red shades, and the second set is shown in blue shades. The n-FUR domains are shown in green and yellow. (A) Top-down view of the TelSAM oligomers. (B) Side view of the TelSAM oligomers. Within the 2D lattice, the interactions between neighboring TelSAM fibers are entirely mediated by the n-FUR domains. Additional figures for the crystal packing are included in the supplemental materials.

The overall crystal packing of the TTT-FUR structure can be described as stacking of 2-D layers to generate a 3 dimensional crystal (Fig 7). The 2-D layer is formed by TelSAM fibers that are interdigitated with the n-FUR domain (Fig 8). Importantly, within this 2-D lattice, the n-FUR domain is the sole contributor to the lateral contacts that connect the TelSAM oligomeric fibers (Fig 8 and S3 File). The stacking of these 2-D layers is mediated by interactions between neighboring TelSAM fibers, as well as interactions between the FUR domains and the TelSAM domains (S1 Fig).

**Fig 9:**
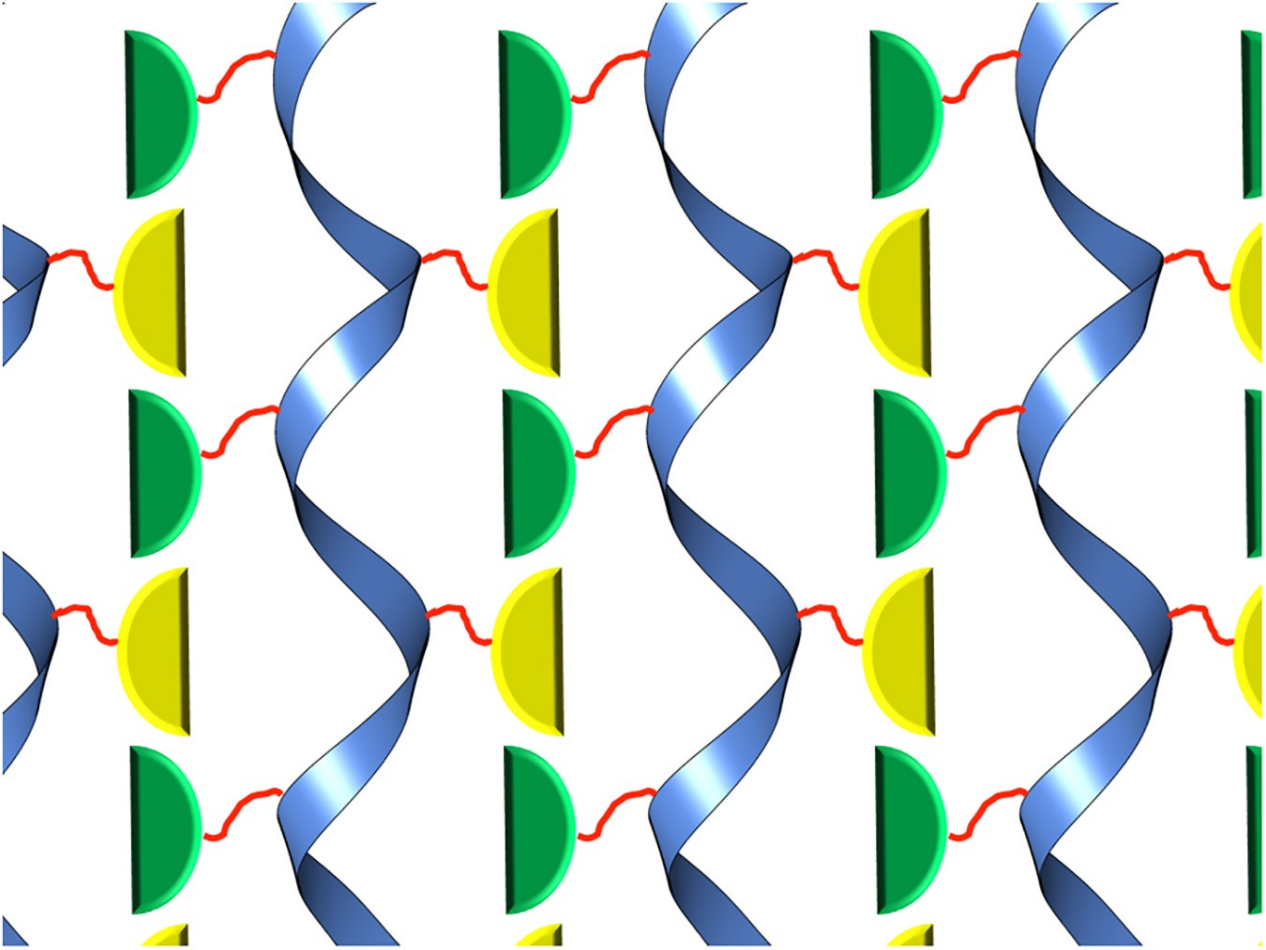
Graphic representation of the observed 2D lattice shown in Fig 8B. The n-FUR domains formed a 2_1_ screw axis instead of a strict 2-fold axis, as shown in Fig 1. The color scheme is the same as used in Fig 1.

Cadmium appeared to lead to quick precipitation of the protein (Fig 3), and the TTT-FUR crystals were obtained in the absence of cadmium. Thus, it was unclear if the FUR domain would display a 2-fold rotational symmetry. Interestingly, instead of forming a strict dimer, the n-FUR domain formed a 2_1_ helical symmetry similar to that of the TelSAM oligomer (Figs 8 and 9). The repeat distance per turn for the n-FUR domain matched the repeat distance per turn for the TelSAM helical oligomer. Both distances equal the unit cell dimension for ‘b’ of 54.8 Å along the Y axis. This probably played an important role in the success in obtaining high quality crystals, and was likely in part due to the similar sizes of the TelSAM and n-FUR domains. The surface area buried between the n-FUR domains is 273.5 Å^2^ for each monomer.

The n-FUR domain attained the desired 2-fold symmetry, although not the packing interactions previously observed (PDB code: 2FU4). However, since the crystals were obtained in the absence of cadmium, it was apparent that the likelihood of obtaining the previously observed dimer [40] was low, and it was unclear which orientation the n-FUR domain would adopt. It is likely that the flexibility of the loops allowed the n-FUR domain to sample a variety of packing interactions and adopt a favorable orientation leading to crystal growth. Interestingly, having flexible loops did not prevent the growth of highly ordered crystals. Instead, the flexibility of the loops likely played an important role in the success of the lattice assembly and it is reasonable to conclude that, as we expected, the lattice assembly is not dependent on having perfect linkers.

## Conclusions

We achieved many of the desired features of the designed 2D lattice. One important feature is that the fusion of three TelSAM domains achieved not only the desired structure, but also the desired property of pH driven oligomerization. This control over the oligomerization process is likely what allowed us to grow good quality 3D crystals. Previously, successful 2D protein lattice designs were only confirmed by EM [28,29]. Thus, here we demonstrated the first 2D lattice design that produced good quality 3D crystals and allowed the structure to be determined to high resolution by X-ray crystallographic methods. Another critically significant feature that was achieved is that the side by side crystal packing interactions between the TelSAM fibers were entirely mediated by the n-FUR domain. This is essential because it suggests that this design is well suited for further exploration for the crystallization of cargo proteins, with the crystallization contacts already prefabricated.

Although the n-FUR domain did not form the previously observed dimer, the crystal packing obtained confirms the validity of our design strategy. Our structure validates that the fusion of three TelSAM domains provides a valid platform for the generation of ordered 2D and 3D lattices. The lattice design described here includes features from what has been described as “rotational symmetry matching”. However, in this case by using a screw axis, we introduced the translational symmetry element to this approach. Translational symmetry can greatly expand the possible patterns that can be achieved by rotational symmetry matching, making it a more powerful and diverse method.

## Supporting information

S1 File Pdb file. Phaser was able to find all four domains. For clarity each domain is labeled as a separate chain.
(PDB)

S2 File Data file. Crystallographic data in mtz format.
(MTZ)

S3 File Two dimensional lattice. Pdb file representing the 2-dimensional protein lattice.
(PDB)

S1 Fig Packing of the TTT-FUR crystals. (A) Top down view of three rows of TTT-FUR. (B) The same three rows shown at a tilt angle. (C) Side view of the same three rows. In All panels the FUR domains are shown in green, and the TelSAM domains are shown in blue and red. The stacking of these 2-D layers is mediated by interactions between neighboring TelSAM fibers, as well as interactions between the FUR domains and the TelSAM domains.
(PDF)
