# Supplementary material for: Symmetry based assembly of a 2 dimensional protein lattice
Source: PLoS One. 2017 Apr 18;12(4):e0174485. doi: 10.1371/journal.pone.0174485 (PMC5395157; doi:10.1371/journal.pone.0174485)

Supplemental Information

Figure S1. Packing of the TTT-FUR crystals.

(**A**) Top down view of three rows of TTT-FUR. (**B**) The same three rows shown at a tilt angle. (**C**) Side view of the same three rows. In All panels the FUR domains are shown in green, and the TelSAM domains are shown in blue and red. The stacking of these 2-D layers is mediated by interactions between neighboring TelSAM fibers, as well as interactions between the FUR domains and the TelSAM domains.

A

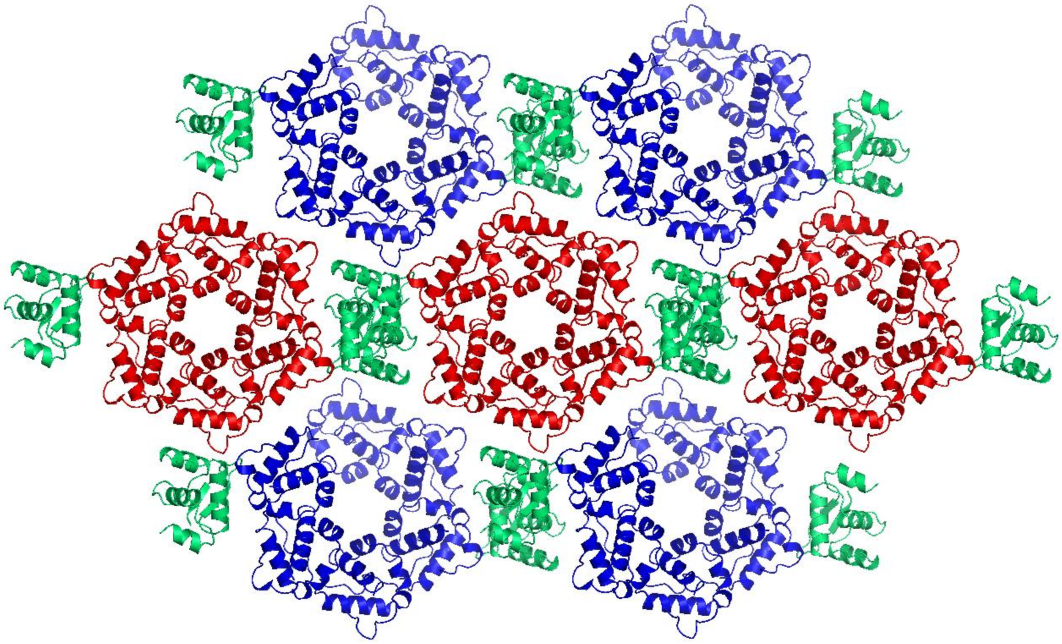

B

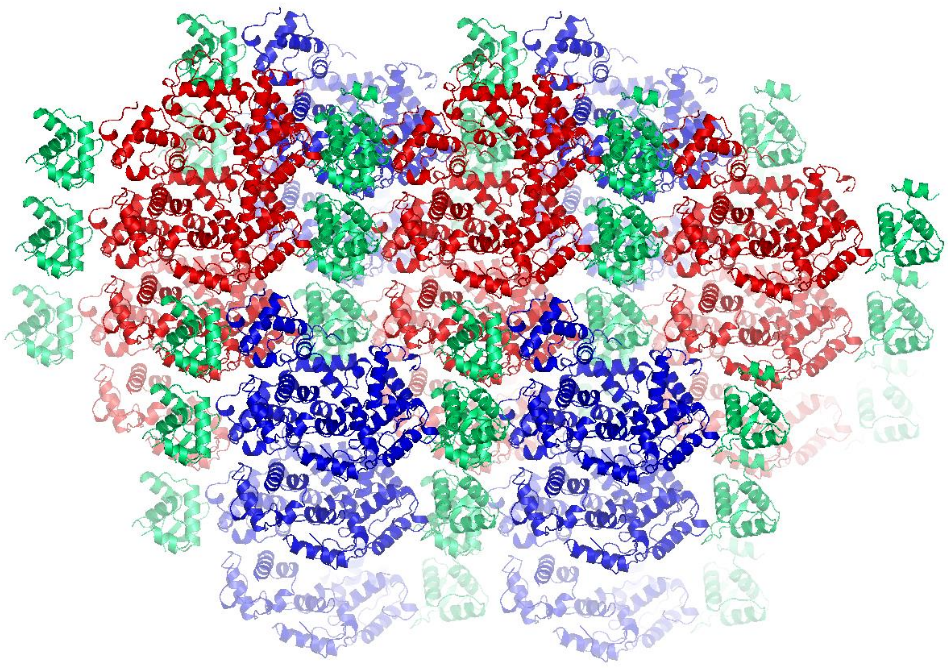

C

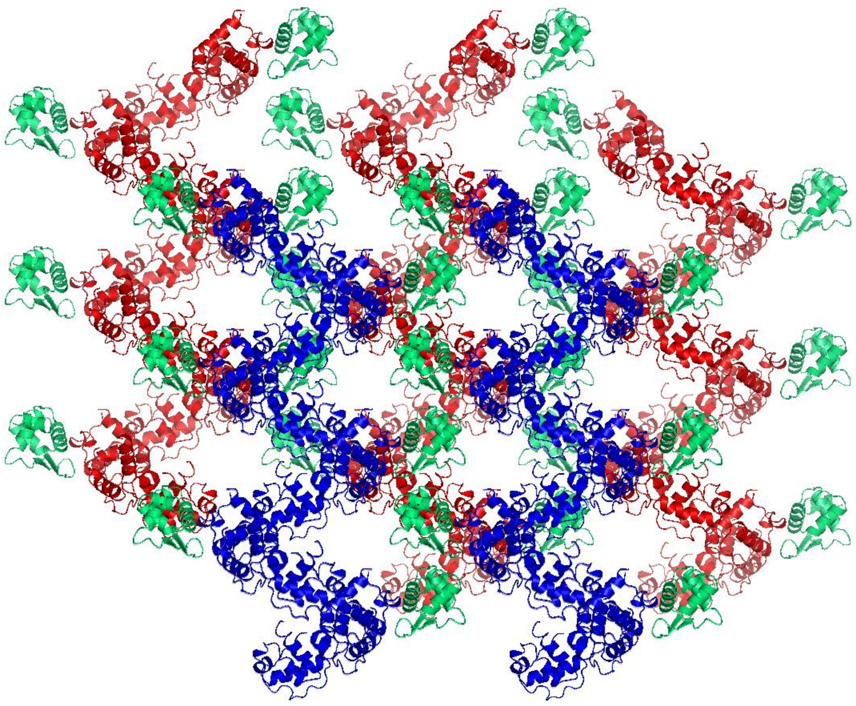

Supplement: S1 Fig — (A) Top down view of three rows of TTT-FUR. (B) The same three rows shown at a tilt angle. (C) Side view of the same three rows. In All panels the FUR domains are shown in green, and the TelSAM domains are shown in blue and red. The stacking of these 2-D layers is mediated by interactions between neighboring TelSAM fibers, as well as interactions between the FUR domains and the TelSAM domains. (PDF) [file pone.0174485.s004.pdf]
